# Metabolic disorders sensitise endometrial carcinoma through endoplasmic reticulum stress

**DOI:** 10.1186/s11658-022-00412-x

**Published:** 2022-12-16

**Authors:** Jingyi Zhou, Yanying Lin, Xiao Yang, Boqiang Shen, Juan Hao, Jiaqi Wang, Jianliu Wang

**Affiliations:** 1 Department of Obstetrics and Gynecology, Peking University People’s Hospital, Beijing, 100044 China; 2 Center of Reproductive Medicine, Fujian Maternity and Child Health Hospital, Affiliated Hospital of Fujian Medical University, Fuzhou, China; 3 Beijing Key Laboratory of Female Pelvic Floor Disorders Disease, Beijing, 100044 China; 4 Fujian Key Laboratory of Women and Children’s Critical Diseases Research, Fujian Maternity and Child Health Hospital, Fuzhou, China

**Keywords:** Endometrial carcinoma, Metabolic disorders, Insulin, Endoplasmic reticulum stress, Saturated fatty acid

## Abstract

**Background:**

Metabolic disorder is considered a well-established risk factor for endometrial carcinoma (EC). However, the mechanism remains unclear. Insulin resistance and excessive flux of free fatty acids serve as fundamental pathogenic factors in metabolic disorders, including obesity and type 2 diabetes. The aim of this study was to test the correlation between insulin resistance and dyslipidaemia in EC and to determine the effect of insulin and saturated fatty acids on EC cells.

**Methods:**

A retrospective study on the medical records of patients with EC and RNA-seq from the TCGA database analysed with edgR and Gene Ontology (GO) were used to assess the correlation of dyslipidaemia and diabetes as well as obesity. Crystal violet assays and CCK-8 assays were used to detect the proliferation of EC cells, and Annexin V-PI was used to examine apoptosis. Transient changes in mitochondrial Ca^2+^ and reactive oxygen species (ROS) were monitored via confocal microscopy. DNA damage was assessed by comet assays. Changes in signalling pathways were detected via phospho-kinase array. western blotting was used to assess the molecular changes in endoplasmic reticulum (ER) stress and DNA damage.

**Results:**

We found that glucose metabolism disorders accompanied dyslipidaemia in patients with EC. As a key regulator of glucose metabolism disorders, insulin promoted DNA damage, ROS and Ca^2+^ homoeostasis imbalance in a panel of established EC cell lines. Interestingly, excessive insulin boosted saturated fatty acid-induced pro-apoptotic effects in EC cells. Furthermore, our data showed that insulin synergised with saturated fatty acids to activate the mechanistic target of rapamycin kinase/70 kDa ribosomal protein S6 kinase (mTOR/p70S6K) pathway and ER stress, resulting in Ca^2+^ release from ER and unfolded protein response (UPR) activation, which contributed to combined insulin and saturated fatty acid treatment-induced apoptosis and tumour progression.

**Conclusions:**

Our data are the first to illustrate that impaired glucose metabolism accelerates dyslipidaemia-promoted EC progression, which is attributed to hyperinsulinaemia and saturated fatty acid-induced Ca^2+^ dyshomoeostasis and UPR activation in EC cells via ER stress.

**Supplementary Information:**

The online version contains supplementary material available at 10.1186/s11658-022-00412-x.

## Background

Endometrial cancer (EC) is the sixth most common malignancy in women and one of the first malignancies to be linked with diabetes and obesity; an estimated 417,000 new cases and 97,000 deaths were estimated in 2020 [1], and 38.4% of EC can be attributed to diabetes combined with obesity [2]. Insulin resistance with excessive flux of saturated fatty acids is considered a fundamental pathogenic factor in metabolic disorders, including obesity and type 2 diabetes [3]. Insulin regulates the growth of endometrial cancer alone [4–6] or in combination with other growth factors [7], but hyperinsulinaemia cannot entirely account for the high risk of EC in individuals with diabetes or obesity. Therefore, the effect of hyperinsulinaemia combined with a high flux of saturated fatty acids/dyslipidaemia on EC progression roused our interest.

Dyslipidaemia is very common in patients with diabetes and obesity. It has been reported that 85.0% of elderly individuals with diabetes have at least one kind of lipid abnormality, and there is a common pattern of lipid abnormalities in individuals with diabetes known as diabetic dyslipidaemia, characterised by increased triglycerides (TGs), reduced high-density lipoprotein (HDL) and a shift towards small dense low-density lipoprotein (LDL) [8]. Obese individuals suffer from adipocyte hypertrophy and excessive adipose tissue accumulation, resulting in abnormal levels of circulating lipids [9]. Insulin resistance is considered the primary cause of dyslipidaemia in diabetes and obesity. Hyperinsulinaemia in insulin resistance increased hepatic production of very-low-density lipoprotein (VLDL), leading to hypertriglyceridaemia and reduced HDL concentration. It was reported that dyslipidaemia, especially hypertriglyceridaemia, increased the risk of EC, but the role of obesity or diabetes cannot be excluded [10]. On the basis of the pathology of dyslipidaemia in metabolic abnormalities, we assumed that dyslipidaemia is closely related to insulin resistance in diabetes and obesity; therefore, excessive insulin and dyslipidaemia may synergistically increase the risk of endometrial cancer.

Hyperinsulinaemia also reduce the suppression of lipolysis in adipose tissue, leading to a high saturated fatty acid flux [11]. It is worth mentioning that TGs can efficiently transport and store saturated fatty acids, which are released as free fatty acids into the circulation after ester hydrolysis of TGs by adipose triglyceride lipase or lysosomal acid lipase [12]. The effect of saturated fatty acids on endometrial cancer remains controversial. It was reported that women with obesity at extreme risk of endometrial cancer have decreased levels of free fatty acids (palmitic acid, stearic acid and oleic acid) after bariatric surgery [13], indicating that serum free fatty acid levels were positively related to EC risk in women with obesity. However, Gaudet et al. found that stearic acid and linoleic acid were lower in EC cases than controls [14]. Fatty acid synthase (FAS), which catalyses the synthesis of PA, modulates the oestrogen receptor to promote EC progression [15], and reduced expression of FAS was observed in progesterone-induced proliferation inhibition in EC cells [16], suggesting that palmitic acid synthesis promotes EC proliferation. However, some free fatty acids, including endogenous ω-3 polyunsaturated fatty acids (ω-3 PUFA) [17], exogenous ω-3 PUFA [18] and lauric acid [19], were found to inhibit proliferation and to promote apoptosis of EC cells. PA is the most abundant free saturated fatty acid in human serum and in the diet [20], and little is known about the effects of PA and its 18-carbon analogue, SA, on EC cells or the effect of insulin combined with saturated fatty acids on EC cells. Therefore, we investigated lipid metabolism- and dyslipidaemia-related gene expression in patients with EC with or without diabetes mellitus and the effect of glucose metabolism disorders combined with saturated fatty acids on EC progression.

ER stress is considered an emerging hallmark of cancer, which may be induced by multiple metabolic and oncogenic abnormalities. Under ER stress, glucose-regulated protein 78 (GRP78), which originally binds to the luminal domain of three ER transducer sensors, i.e. protein kinase RNA-like ER kinase (PERK), inositol-requiring protein 1 (IRE1) and activating transcription factor 6 (ATF6), dissociates from these three ER transducer sensors. Persistent ER stress may ultimately drive the activation of unfolded protein response (UPR), which is involved in metastasis and angiogenesis of cancers [21].

In this study, we found that insulin, a key regulator of impaired glucose metabolism, accelerates saturated fatty acid-promoted apoptosis through mechanistic target of rapamycin kinase/70 kDa ribosomal protein S6 kinase (mTOR/p70S6K) pathway activation and ER stress-mediated Ca^2+^ dyshomoeostasis. Our data revealed that excessive insulin acts as an important factor in promoting lipid metabolic disorder-induced EC progression and might be a potential therapeutic target in patients with EC complicated with metabolic disorders.

## Methods

### Clinical data analysis

A retrospective study of blood glucose and serum lipids of 295 patients with newly diagnosed EC using the medical records of patients diagnosed from January 2010 to December 2018 was conducted. Patients were identified from the electronic medical database of Peking University People’s Hospital (Beijing, China). Clinical characteristics, including age, body mass index (BMI), menopause and FIGO stage, were obtained. Pre-treatment, fasting glucose and serum lipids, including triglyceride (TG), low-density lipoprotein (LDL), high-density lipoprotein (HDL), and total cholesterol (TC) values, were extracted. This study was approved by the institutional review board of Peking University People’s Hospital (2015PHB116-01).

### TCGA analysis

The Cancer Genome Atlas (TCGA) sponsored by the National Cancer Institute is a publicly available resource in which multidimensional cancer genomics and clinical datasets are deposited. We downloaded UCEC level 3 genomics data (RNAseqV2) from the TCGA data portal (https://tcga-data.nci.nih.gov/tcga/), and clinical information was downloaded from Firehose (http://gdac.broadinstitute.org/). The data used in this study were updated as of 27 October 2018. Individuals with UCEC that had a history of diabetes were regarded as UCEC with diabetes, while individuals with UCEC but without a history of diabetes were considered UCEC without diabetes. A total of 420 patients were analysed, including 117 patients with UCEC with diabetes and 303 patients with UCEC without diabetes.

Raw counts of gene expression from RNA-seq deposited in the TCGA data portal were used for the differential gene expression. The analysis was performed through the edgeR package in R [21], and the differential gene expression was examined by accounting for variability through an overdispersed Poisson model and moderating the degree of overdispersion by empirical Bayes methods [22]. In this study, counts per million (CPM) was calculated through the program, and only genes with CPM values larger than 0.5 across at least two samples were considered. A generalised linear model plus likelihood ratio test was used to calculate the significance as well as the fold change (FC). The genes were considered statistically significant when the adjusted *P* value was less than 0.05 and the absolute FC was larger than 1.5.

Gene Ontology (GO) analysis is a useful method for annotating genes and gene sets with biological characteristics for high‑throughput genome or transcriptome data [23]. GO analysis was performed using the Enrichr tools available at the Enrichr Web site (https://maayanlab.cloud/Enrichr/) [24].

### Cell culture, reagent and inhibitors

Hec1A (lot no. 58087755), RL952 (lot no. 62130010) and AN3CA (lot no. 62130010) human EC cell lines were purchased from the American Type Culture Collection (ATCC, Manassas, USA). The human EC cell line Ishikawa was obtained from our laboratory stock. The human endometrial cancer cell lines RL-952 and Ishikawa were maintained in Dulbecco’s modified Eagle medium/Nutrient Mixture F-12 (DMEM/F12) (SH30023.01B, HyClone, USA) supplemented with 10% foetal bovine serum (FBS; 16000044, Gibco, USA) and 1% penicillin/streptomycin (CC004, M&C GENE, China). HEC-1B and AN3CA cells were maintained in MEM (CM10010, M&C GENE, China) supplemented with non-essential amino acids (11140076, Gibco, USA), 1 mM sodium pyruvate (11360070, Gibco, USA), 10% FBS and 1% penicillin/streptomycin. All the cells were incubated in a humidified atmosphere with 5% CO_2_ at 37 °C. Insulin (I0516) was obtained from Sigma-Aldrich. ATF6α inhibitor Ceapin-A7 (HY-108434), PERK inhibitor ISRIB (HY-12495) and IRE1 inhibitor 4μ8C (HY-19707) were purchased from MCE (MedChemExpress, USA). mTOR siRNA sequences were: 1# “CCA CCC GAA UUG GCA GAU UTT”, “GCA AAG AUC UCA UGG GCU UTT”. To measure glucose uptake, a fluorescent indicator 2-[*N*-(7-nitrobenz-2-oxa-1, 3-diazol-4-yl)amino]-2-deoxy-*D*-glucose (2-NBDG) (B6035) was purchase from Apexbio (APExBIO Technology LLC, USA).

### Preparation of saturated fatty acids

NaOH and PA (#P9767, Sigma-Aldrich, USA) or SA (#S3381, Sigma-Aldrich, USA) were prepared to an equimolar concentration of 100 mM and heated in a 90 °C water bath until the solution was clear to dissolve saturated fatty acids. A 15% (wt/vol) BSA solution was prepared in 0.09% NaCl solution. The prepared 100 mM NaOH-PA or 100 mM NaOH-SA solution was added dropwise to the 15% BSA solution in a water bath at 55 °C, vortexed for 10 s and incubated at 55 °C for 10 min. The saturated fatty acid-BSA complex solution was cooled to room temperature and filtered with a 0.45-μm sterile filter. The saturated fatty acid–BSA stock solution was diluted to the working concentration in medium before use.

### CCK-8 cell proliferation assay

We incubated 1000 EC cells (Ishikawa, RL952 and Hec1A) with different glucose and insulin concentrations (2.5 mM or 25 mM glucose, with 0, 10 or 100 nM insulin) in 96-well plates for 4 consecutive days. Then, we added 10 μL of Cell Counting Kit-8 solution (CCK-8; CK04, Dojindo, Japan) to each well and measured the colour intensity in a microplate reader (Tecan Infinite 200) at 490 nm. Experiments were performed in triplicate.

### Apoptosis

Cells were treated with vehicle or 100 nM insulin and incubated in 5% CO_2_ at 37 °C for 3 h or 24 h. The apoptotic population of cells was analysed with an Annexin V-FITC Apoptosis Detection Kit I (#556547, BD Bioscience, USA) according to the manufacturer’s instructions and processed by flow cytometry (FACSCanto II, BD Bioscience, USA).

### Western blotting

Total protein lysates were prepared from EC cells and quantified by the Bradford method (5000201, Bio-Rad, USA). Equal amounts of cell lysates (20–40 μg) were separated by 6–15% SDS‒PAGE and transferred onto nitrocellulose membranes (Merck Millipore Ltd) (1.5–2 h run time; 2 h to transfer). Then, the blots were blocked with 5% non-fat milk and incubated with primary antibodies against Bax (1:1000, #2774, Cell Signaling, USA), Bcl-2 (1:1000, # 15071, Cell Signaling, USA), γ-H2AX (1:1000, #9718, Cell Signaling, USA), p-Chk1 (1:1000, #2348, Cell Signaling, USA), p-ATR (1:1000, #2853, Cell Signaling, USA), CHOP (1:1000, #2895, Cell Signaling, USA), GRP78 (1:1000, #3177, Cell Signaling, USA), p-eIF2α (1:1000, #3597, Cell Signaling, USA), p-p70S6K (1:1000, #9234, Cell Signaling, USA), mTOR (1:1000, #2972, Cell Signaling, USA), β-actin (1:2000, #4970, Cell Signaling, USA) and GAPDH (1:2000, #2118, Cell Signaling, USA) at 4 °C overnight. Finally, the blots were incubated with HRP-conjugated goat anti-rabbit secondary antibody (ZB2301, ZSGB-BIO, Beijing) for 2 h at room temperature and developed with ECL (P1020, Applygen, China). The band intensities were determined with the Bio-Rad imaging system (Hercules, CA, USA).

### Comet assay

Briefly, 1 × 10^6^ cells/mL were treated with either vehicle or 100 mM insulin and incubated in 5% CO_2_ at 37 °C for 3 h. After incubation, the cells were centrifuged and washed with cold PBS, and 1 × 10^5^ cells/mL based on counts from the pool of untreated and treated cells were used for performing the comet assay as previously described and stained with PI.

### Human phospho-kinase antibody array

The Human Phospho-Kinase Array Kit (#ARY003B) was purchased from R&D Systems (Minneapolis, MN). Array screening was performed following the manufacturer’s protocol. Briefly, cell lysates were incubated with the array membranes. After washing, the membranes were incubated with biotinylated antibody cocktail. The amounts of phosphokinase were assessed with streptavidin conjugated to horseradish peroxidase, followed by chemiluminescence detection. A Bio-Rad imaging system (Hercules, CA, USA) was used to quantify the density of each dot against the average of the internal controls on the membrane as indicated in the protocol.

### Measurement of mitochondrial Ca^2+^ concentrations

Ishikawa cells grown on glass-bottom Petri dishes (Solarbio, China, # YA0570) were loaded with 2 μM Rhod-2/AM (#R1244, Invitrogen, USA) and MitoTracker Green (#M7514, Invitrogen, USA) for 30 min in Hank’s balanced salt solution (#CC025, M&C GENE, China). After 60 s of baseline image recording, insulin was added to the Petri dish to a final concentration of 10 nM, and confocal images were recorded for 10 min by a laser-scanning confocal microscope (TCS SP8; Leica, Germany) at 488-nm and 561-nm excitation using a 20× objective lens.

### Real-time PCR analysis of mRNA

Real-time PCR analysis of mRNA. Total RNA was isolated with TRIzol reagent (Invitrogen, 15596-026). cDNA was synthesised from 2 μg of RNA with the use of the Quantscript RT Kit (TianGen, KR118). Primer sequences of APOB, APOA2, APOC3, ACTB for RT-PCR were as follows: APOB, TGT CAG TAC ACA CTG GAC GC, TCA AAT GCG AGG CCC ATC TT; APOA2, GTC AAG AGC CCA GAG CTT CA, GCT GTG TTC CAA GTT CCA CG; APOC3, ACC CTG AGG TCA GAC CAA CT, AGG AGC TCG CAG GAT GGA TA; ACTB, CCA CCA TGT ACC CTG GCA TT, CGG ACT CGT CAT ACT CCT GC.

### Statistics

Descriptive statistics are presented as percentages or means ± standard deviations (SDs). All tests were two-sided, and a *P* value < 0.05 was considered statistically significant. Statistical analyses were performed using SPSS 20.0 software (IBM Corporation, Armonk, NY, USA).

## Results

### Glucose metabolism disorders were accompanied by dyslipidaemia in patients with EC

**Fig. 1:**
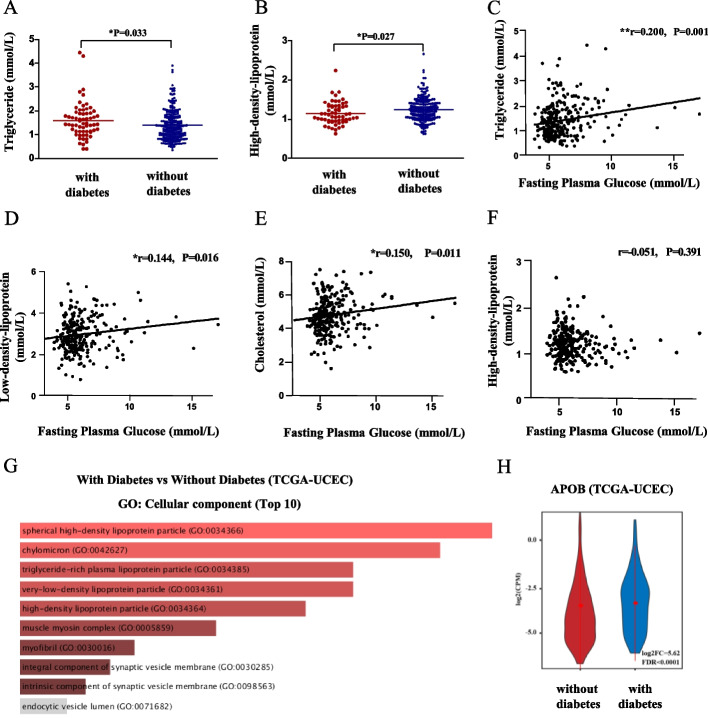
Endometrial cancer patient with diabetes mellitus accompanied with lipid metabolism disorder. **A**, **B** The levels of serum lipids in diabetic and non-diabetic patients with EC. Triglyceride (TG) was higher in diabetic patients with EC than non-diabetic patients with EC, *P* = 0.033. High-density lipoprotein (HDL) was significantly lower in diabetic patients than non-diabetic patients, *P* = 0.027. **C**–**F** The correlation of fasting plasma glucose (FPG) and serum lipids. TG, low-density lipoprotein (LDL) and cholesterol (CHO) were positively correlated with FPG. The correlation coefficient *r* and *P* value are shown. **G** Cellular component Gene Ontology (GO) analysis of the differential expression genes (DEGs) in EC with and without diabetes. DEGs were analysed using Enrichr database, on the basis of adjusted *P* value (*Q* value) ranking. The brighter the colour, the more significantly enriched the GO term displayed. **H** Expression levels of APOB in patients with EC with or without diabetes. In the violin plot, the red dot represents the median, the red line represents the 95% confidence interval

To further investigate the relationship between glucose metabolism disorder and dyslipidaemia, we retrospectively assessed the clinical characteristics of patients diagnosed with EC from Peking University People’s Hospital. A total of 295 EC cases were reviewed, and the demographic characteristics are shown in Additional file 1: Table S1. We found that triglycerides (TGs) were higher in diabetic patients with EC than in non-diabetic patients with EC (1.59 ± 0.74 mmol/L versus 1.39 ± 0.63 mmol/L, *P* = 0.033), while high-density lipoprotein (HDL) was significantly lower in diabetic patients with EC than in non-diabetic subjects (1.15 ± 0.28 mmol/L versus 1.25 ± 0.31 mmol/L, *P* = 0.027) (Fig. 1A and B). There were no significant differences in low-density lipoprotein (LDL) (*P* = 0.193) or total cholesterol (CHO) (*P* = 0.262) between the two groups (Additional file 1: Fig. S1A and B). In addition, we found that TG, LDL and CHO were all positively correlated with fasting plasma glucose (FPG), and the correlation coefficients were 0.200 (*P* = 0.001) for TG, 0.144 (*P* = 0.016) for LDL and 0.150 (*P* = 0.011) for CHO (Fig. 1C, D and E). However, we did not find a significant correlation between HDL and FPG (Fig. 1F). We further investigated the relationship between serum lipids and BMI. The TG levels were 1.61 ± 0.63 mmol/L in the obese (BMI ≥ 30 kg/m^2^) patients with EC and 1.40 ± 0.68 mmol/L in the nonobese (BMI < 30 kg/m^2^) patients with EC, and the difference was considered to be borderline significant with *P* = 0.064. The difference in HDL between obese and nonobese patients with EC was also considered to be borderline significant (1.15 ± 0.32 mmol/L versus 1.24 ± 0.30 mmol/L, *P* = 0.076). No significant difference was found in LDL (*P* = 0.459) or CHO (*P* = 0.507) between obese and nonobese subjects with EC (Additional file 1: Fig. S1C-F). Interestingly, we found a significant positive correlation between TG and BMI (*r* = 0.142, *P* = 0.019) and a negative correlation between HDL and BMI (*r* = − 0.127, *P* = 0.001), while no correlation between BMI and CHO or LDL was detected (Additional file 1: Fig. S1G and H). These results demonstrated the change in dyslipidaemia-associated characteristics in patients with EC with impaired glucose metabolism or high BMI.

Next, we investigated the differences in lipid regulation-related genes between diabetic and non-diabetic individuals with EC through TCGA. We analysed the differentially expressed genes (DEGs) in EC with and without diabetes, and 399 DEGs were obtained. We found that the most significantly regulated DEGs were enriched in lipoprotein particles in the cellular components category, especially in TG-rich plasma lipoprotein particles and very-low-density lipoprotein particles, which carry abundant TGs (Fig. 1G). For biological processes, the most significantly regulated DEGs of diabetic EC patients were enriched in chylomicron remodelling and assembly. In the molecular function category, the most significantly upregulated DEGs were enriched in lipoprotein particle receptor binding (Additional file 1: Fig. S2A). Furthermore, we observed that several apolipoprotein genes were upregulated in diabetic patients with EC. APOB, encoding apolipoprotein B, the main apolipoprotein of chylomicrons and LDLs, was highly upregulated in patients with EC with diabetes, with an FC of 38.3 (FDR < 0.001) (Fig. 1H). APOA2, encoding apoA-II, which is the second most abundant apolipoprotein of HDL particles, was upregulated in diabetic subjects (FDR < 0.001; FC: 48.2) (Additional file 1: Fig. S2B). APOC3, encoding apoC3, was overexpressed in EC with diabetes with an FC of 5.35 (FDR < 0.001) (Additional file 1: Fig. S2C). To further validate the expression (NGS) data, we performed real-time PCR using six EC samples. Three of the six EC samples were from patients with diabetes, and three were from patients without diabetes. The expression levels of genes in “without” groups were set to 1 and the expression levels of genes in “with” groups were reported as fold change relative to “without” groups. qPCR result confirmed that APOB, APOA2 and APOC3 were significantly upregulated in “with” groups compared with “without” groups (Additional file 1: Fig. S2D). These findings indicated that changes in plasma lipid regulation were one of the most prominent characteristics of diabetic patients with EC.

### Excessive insulin, the key regulator of glucose metabolism disorders, promotes DNA damage, ROS and Ca^2+^ homoeostasis imbalance in EC cells

To investigate the effect of glucose metabolism disorders on patients with EC, we treated EC cells with insulin, one of the key regulators of hyperglycaemia, and studied the biological behaviour changes in cells. We first performed a CCK-8 assay to evaluate the proliferation of the Ishikawa, HEC-1B and RL952 cell lines that were treated with different doses (0, 1, 10 and 100 nM) of insulin for 0–72 h, and no changes in the proliferation of EC cell lines in the insulin-treated groups were detected (Additional file 1: Fig. S3A). To rule out the interference of FBS, we further investigated the effects of 10 nM insulin with different concentrations of FBS (0%, 1%, 2.5%, 5% and 10%) and found that insulin treatment did not affect the proliferation of Ishikawa cells (Additional file 1: Fig. S3B). Therefore, we concluded that insulin per se does not promote proliferation in EC cells.

We further investigated whether insulin affects apoptosis of EC cells. We found that, after treatment with 100 nM insulin for 24 h, there was no difference in apoptosis between the vehicle group and the insulin treatment group in either Ishikawa (2.040 ± 0.691% versus 1.653 ± 0.633%, *P* = 0.7009) or Hec-1B cells (2.100 ± 0.608% versus 2.333 ± 0.521%, *P* = 0.7852) (Additional file 1: Fig. S4A). However, insulin treatment affected the expression of Bax and Bcl-2 protein in both Ishikawa and Hec-1B cells. For Ishikawa cells treated with insulin for 24 h, the expression of Bax protein increased gradually and then decreased, and the highest expression of Bax protein was observed at 6 h of insulin treatment. The expression of Bcl-2 decreased with insulin treatment. Moreover, the maximum Bax/Bcl-2 ratio was observed at 6 h of insulin treatment. For HEC-1B cells, the expression of Bax protein increased with prolonged insulin treatment within 24 h, while the expression of Bcl-2 did not change significantly; therefore, the Bax/Bcl-2 ratio reached a maximum at 24 h (Additional file 1: Fig. S4B).

**Fig. 2:**
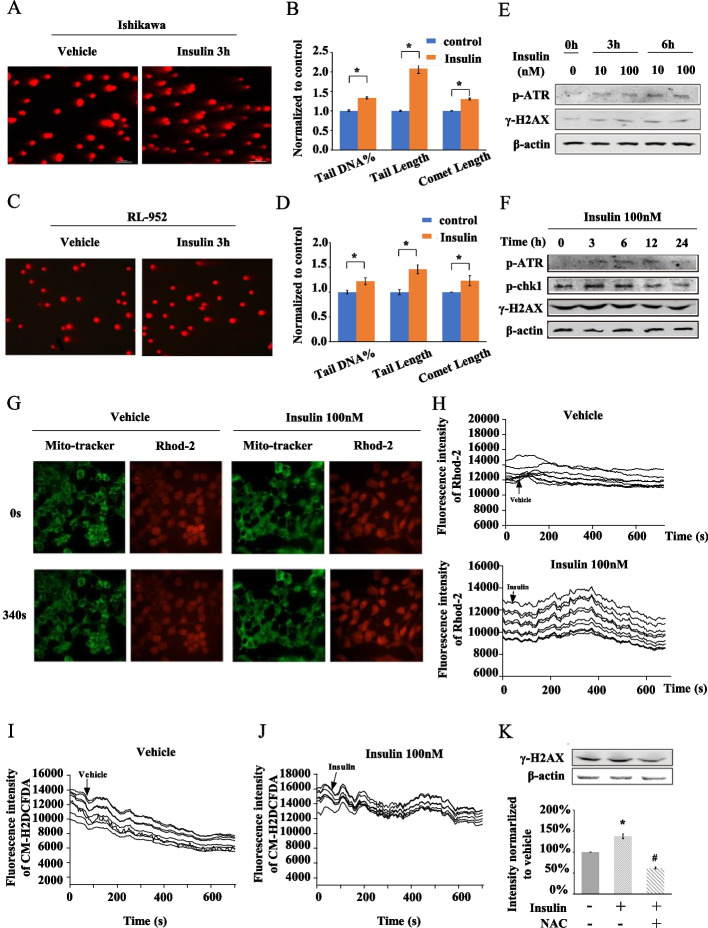
Insulin induces DNA damage, ROS and Ca^2+^ homoeostasis imbalance. **A**, **C** Comet images assessing DNA damage in Ishikawa cells and RL-952 cells after treatment with insulin (100 nM) for 3 h. **B**, **D** Statistical analysis of tail DNA, tail length and comet length by comet assay software. *n* = 100. **E** Immunoblot of DNA damage repaired response kinase p-ATR and DNA damage marker γ-H2AX in Ishikawa cells after insulin (10, 100 nM) treatment for 0, 3 and 6 h. **F** Immunoblot of p-ATR, p-Chk1 and γ-H2AX in Ishikawa cells after insulin (100 nM) treatment for 0, 3, 6, 12 and 24 h. **G** Confocal microscopy detecting transient changes of mitochondrial Ca^2+^ after insulin (100 nM) stimulation. (200×) Ishikawa cells are loaded with mitochondrial probe Mito-tracker and mitochondrial Ca^2+^ probe Rhod-2, then stimulated by vehicle or insulin. **H** Fluorescence intensity indicating mitochondrial Ca^2+^ concentration change within 600 s treatment. Each curve indicates the change in a single isolated cell. **I**, **J** ROS analysis in Ishikawa cells after stimulation by vehicle or insulin (100 nM). CM-H2DCFDA is ROS indicator. **K** Immunoblot of γ-H2A in Ishikawa cells after insulin and/or NAC treatment. Histogram plot shows γ-H2A expression level relative to β-actin. *P < 0.05, insulin treatment alone compared with vehicle. #*P* < 0.05, NAC + insulin combination treatment compared with insulin treatment alone

Upregulated expression of Bax and downregulated expression of Bcl-2 could be triggered by ATR-Chk1 or ATM-Chk2 activation in the DNA damage response [25]. Therefore, we further investigated whether insulin induced DNA damage in EC cells. In Ishikawa cells, we performed a comet assay to detect DNA damage and found that, after insulin treatment for 3 h, Ishikawa cells showed more significant tailing than that of vehicle-treated cells (Fig. 2A). After analysing 100 cells from each group by CASP software, we found that insulin-treated Ishikawa cells had significantly more tail DNA (*P* = 0.0028), a longer tail length (*P* < 0.0001) and a longer comet length (*P* < 0.0001) (Fig. 2B). Similar results were also observed in RL-952 cells (Fig. 2C and D). Furthermore, we found that 100 nM insulin increased the phosphorylation of γ-H2AX (Ser139), as well as the phosphorylation of p-ATR (Ser428) and p-Chk1 (Ser345), and the effect was pronounced at 3–6 h (Fig. 2E and F). These results indicated that insulin induced DNA damage in EC cells.

Furthermore, we examined the mitochondrial Ca^2+^ dynamics and reactive oxygen species (ROS) change after insulin stimulation in Ishikawa cells by confocal microscopy and found that, compared with the vehicle, the mitochondrial Ca^2+^ and ROS of EC cells increased within 10 min after insulin stimulation. The peak of mitochondrial Ca^2+^ was observed approximately 300–400 s after insulin stimulation, and the peak of ROS production was approximately 400–500 s after insulin stimulation (Fig. 2G–J). To investigate whether the ROS increase is the root of insulin-induced DNA damage, we used acetylcysteine (*N*-acetyl-l-cysteine, NAC) to inhibit ROS in EC cells and found that, after inhibiting ROS, insulin-induced DNA damage was decreased (Fig. 2K). Therefore, our results illustrated that, as a key regulator of glucose metabolism disorders, insulin increased DNA damage, ROS and Ca^2+^ homoeostasis imbalance in EC cells.

### Insulin boosts saturated fatty acid-induced apoptosis in EC cells

**Fig. 3:**
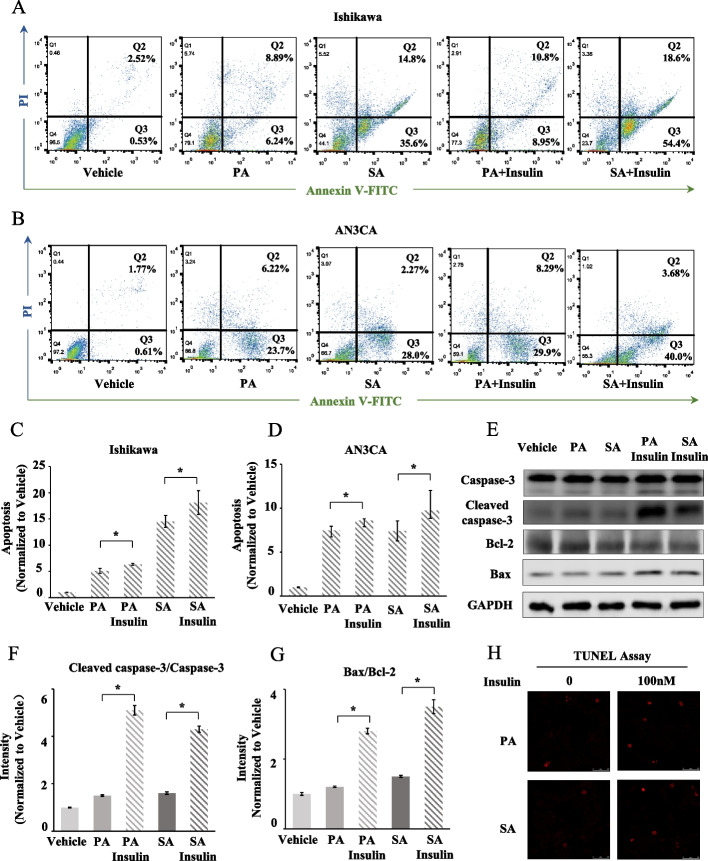
High insulin level accelerates palmitic acid-induced apoptosis in endometrial cancer cells. **A**, **B** Apoptosis of Ishikawa and AN3CA cells detecting by Annexin V-FITC/PI labelling flow cytometry after saturated fatty acid and/or insulin treatment. The proportion of apoptotic cells was detected after treatment with 0.5 mM palmitic acid (PA) or 0.5 mM stearic acid (SA) for 12 h in Ishikawa cells and treatment for 18 h in AN3CA cells. The proportion of apoptotic cells were also compared after treatment with 0.5 mM PA alone or 0.5 mM PA plus 100 nM insulin, 0.5 mM SA alone or 0.5 mM SA plus 100 nM insulin. Saturated fatty acid (PA and SA) significantly induced apoptosis in EC cells, and the pro-apoptotic effect could be enhanced by excessive insulin. **C**, **D** Analysis of Annexin-V-positive cell ratio *P < 0.05. **E**–**G** Immunoblot of caspase-3, cleaved caspase-3, Bcl-2, Bax and GAPDH after saturated fatty acid and/or insulin treatment. Ishikawa cells are treated with 0.5 mM PA or 0.5 mM SA alone, and PA or SA plus 100 mM insulin for 12 h. Histogram plot shows cleaved caspase-3/caspase-3 and Bax/Bcl-2 intensity relative to vehicle *P < 0.05. **H** Apoptotic effect of insulin on PA or SA-treated (0.5 mM) Ishikawa cells, determined by TUNEL assay (red)

To investigate the combined effect of glucose metabolism disorders and dyslipidaemia on EC, we studied the synergistic effect of insulin and saturated fatty acids on EC cells. We found that the apoptotic population of Ishikawa cells was significantly increased with 0.5 mM PA treatment for 12 h (*P* < 0.0001) and 0.5 mM SA treatment for 12 h (*P* < 0.0001). Moreover, with the addition of 100 nM insulin, the apoptotic population of Ishikawa cells was more obvious than that with PA treatment alone (*P* = 0.0416) or SA treatment alone (*P* = 0.0397) (Fig. 3A and C). Similar results were also observed in AN3CA cells; 0.5 mM SA or PA treatment for 18 h induced apoptosis in AN3CA cells (*P* < 0.0001 for PA and *P* = 0.0006 for SA), with AN3CA cells treated with insulin combined with PA for 18 h showing a greater apoptotic population than those treated with PA alone (*P* = 0.0295) and treatment with insulin combined with SA for 18 h inducing more apoptosis than PA alone (*P* = 0.0480) (Fig. 3B and D).

Moreover, we performed western blotting and TUNEL assays to assess the extent of insulin-saturated fatty acid combination treatment-induced cell apoptosis. The data showed that the ratios of cleaved caspase-3/caspase-3 and Bax/Bcl-2 were significantly reduced in the insulin-saturated fatty acid combination groups compared with the saturated fatty acid treatment alone groups (*P* < 0.0001), and apoptotic cells stained by TUNEL were increased (Fig. 3E–H). These data illustrated that insulin boosts saturated fatty acid-induced apoptosis in EC cells, which results in dysfunction of apoptosis and may finally mediate EC progression.

### Insulin and saturated fatty acids synergistically promote cell apoptosis and Ca^2+^ homoeostasis imbalance through endoplasmic reticulum stress

**Fig. 4:**
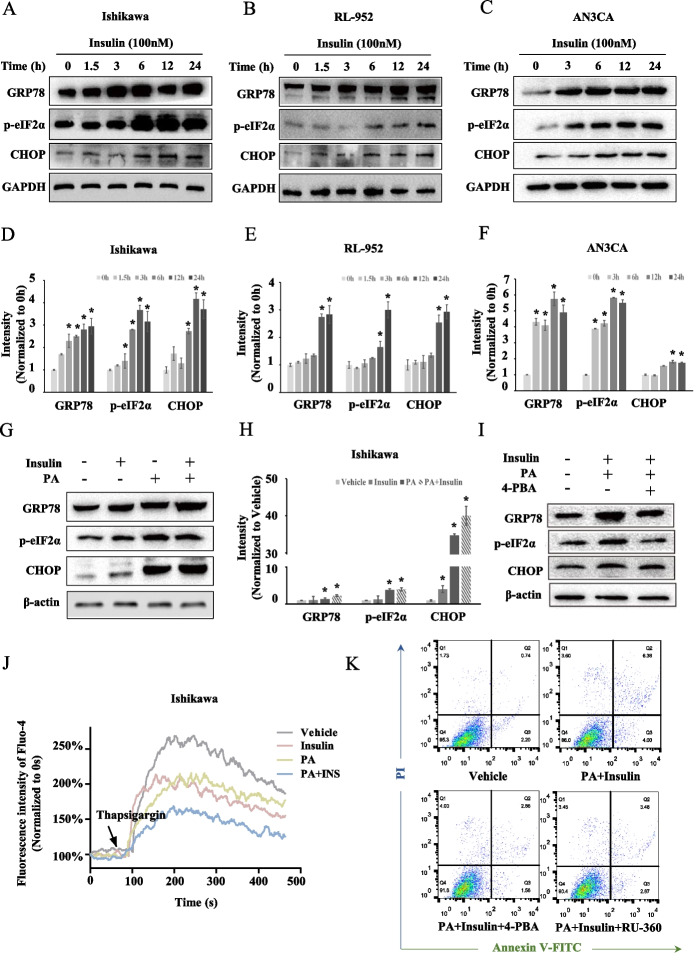
Endoplasmic reticulum stress and Ca^2+^ homoeostasis are essential for promoting apoptosis by combined effect of insulin and palmitic acid. **A**–**C** Immunoblot of ER stress marker GRP78, p-eIF2α and CHOP after treating with 100 nM insulin for 0–24 h in Ishikawa, RL-952 and AN3CA cells. **D**–**F** Histogram plot shows change of ER stress marker with insulin treatment in EC cells relative to 0 h. *P < 0.05. **G**, **H** Immunoblot of ER stress marker GRP78, p-eIF2α and CHOP after treating with insulin, PA and insulin plus PA. Histogram plot shows change of ER stress marker with insulin and/or PA treatment *P < 0.05. **I** Immunoblot of GRP78, p-eIF2α and CHOP after PA plus insulin treatment with or without 4-phenylbutyric acid (4-PBA) pre-treatment. **J** Ca^2+^ store in endoplasmic reticulum after treatment with vehicle, 100 nM insulin alone, 0.5 mM PA alone or 0.5 mM PA plus 100 nM insulin for 6 h. **K** Apoptosis of Ishikawa cells detecting by Annexin V-FITC/PI labelling flow cytometry after 0.5 mM PA plus 100 nM insulin treatment for 12 h, with or without pretreatment with 4-phenylbutyric acid (4-PBA) or RU-360

We next investigated the mechanism by which insulin promotes saturated fatty acid-induced apoptosis. According to our data, excessive insulin reduced mitochondrial Ca^2+^ homoeostasis. The release of Ca^2+^ from ER stores has been implicated as being directly responsible for mitochondrial overload in multiple models of apoptosis [26, 27]. As the activation and persistence of endoplasmic reticulum (ER) stress is accompanied by the release and depletion of Ca^2+^ stores in the ER, we further explored whether ER stress is involved in the synergistic effect of insulin and saturated fatty acids on EC cells. First, we found that insulin induced ER stress in EC cells. In Ishikawa, RL-952 and AN3CA cell lines, 100 nM insulin, in a time-dependent manner, induced increases in GRP78 and transcription factor C/EBP homologous protein (CHOP) expression level and the upregulation of p-eIF2α (Ser51) within 24 h (Fig. 4A–F). Furthermore, 0.5 mM PA alone also upregulated GRP78, CHOP and p-eIF2α (Ser51) in Ishikawa cells. Interestingly, when EC cells were treated with PA combined with 100 nM insulin for 12 h, the expression of GRP78, CHOP and p-eIF2α (Ser51) was significantly upregulated compared with PA treatment alone or insulin treatment alone, indicating that insulin and saturated fatty acids presented synergistic effects in inducing ER stress in EC cells (Fig. 4G and H). Next, we treated cells with the ER stress inhibitor 4-phenylbutyric acid (4-PBA) to assess the role of ER stress in the apoptosis induced by the combination of insulin and saturated fatty acid treatment. Insulin combined with PA-induced ER stress was decreased by 4-PBA pre-treatment, and pre-treatment of Ishikawa cells with 4-PBA abolished apoptosis induced by PA combined with insulin (7.83 ± 0.21% versus 4.88 ± 0.40%, *P* = 0.0029) (Fig. 4I, K).

We explored the combination treatment effect on the ER Ca^2+^ pool on the basis of the finding that insulin and saturated fatty acids synergistically induced ER stress. ER stress is involved in the release of ER Ca^2+^, which can be indirectly detected as the ER Ca^2+^ stores with Fluo-4 cytosolic staining after stimulation with the ER stressor thapsigargin. The data showed that after 6 h of treatment with insulin alone, PA alone or PA combined with insulin, the Ca^2+^ stores in the ER were all decreased in EC cells, and the most significant decrease was observed in cells treated with PA combined with insulin (Fig. 4J). The data illustrated that, accompanying ER stress, Ca^2+^ was released from the ER and may result in mitochondrial Ca^2+^ overload.

We further investigated whether the increase in ER stress and increased uptake of mitochondrial Ca^2+^ were involved in apoptosis of EC cells induced by saturated fatty acids combined with insulin. With pre-treatment of Ishikawa cells with 4-PBA, cell apoptosis was decreased (Fig. 4K). Pre-treatment of Ishikawa cells with RU-360, a cell-permeable oxygen-bridged dinuclear ruthenium amine complex mitochondrial Ca^2+^ uptake inhibitor, to inhibit mitochondrial Ca^2+^ uptake also abolished the apoptotic population induced by PA combined with insulin (7.83 ± 0.21% versus 5.63 ± 0.29%, *P* = 0.0034) (Fig. 4K). These results indicate that ER stress and disordered Ca^2+^ mediate apoptosis caused by saturated fatty acids combined with insulin in EC cells. Therefore, ER stress plays an important role in the cell apoptosis and Ca^2+^ homoeostasis synergistically induced by insulin and saturated fatty acid combined treatment.

### Activation of the mTOR/p70S6K pathway is key for metabolic disorder-induced ER stress

**Fig. 5:**
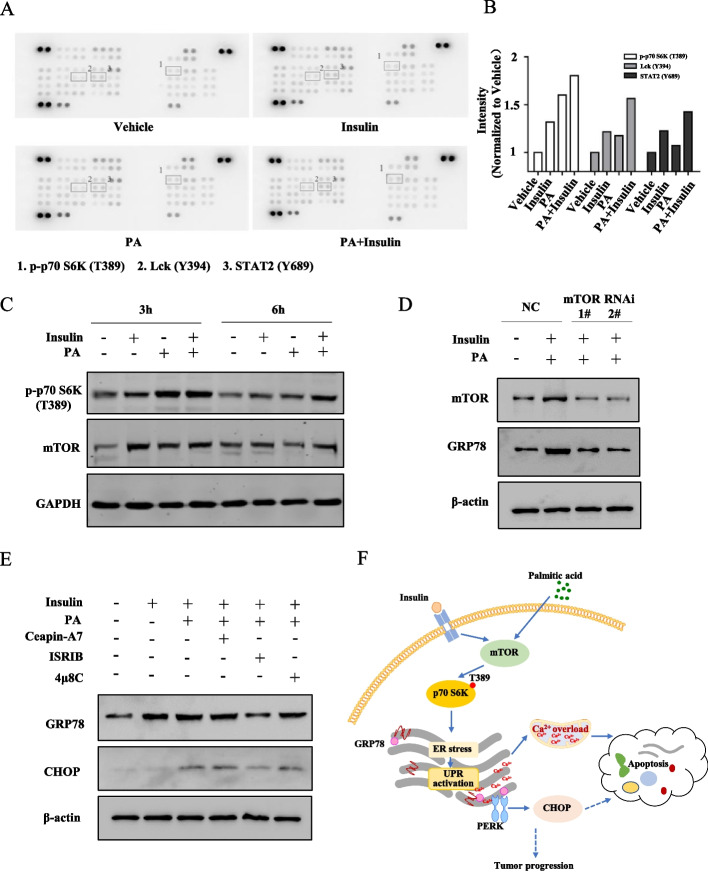
mTOR/p70S6K is the key for metabolic-disorder-induced endoplasmic reticulum stress. **A** Phosphoproteomic analysis of insulin and/or PA-stimulated Ishikawa cells. After treatment with vehicle, 100 nM insulin, 0.5 mM PA or 0.5 mM PA plus 100 nM insulin for 3 h, the expression levels of phosphorylation sites were assessed by phospho-kinase array. Signals of relevant kinases in response to insulin and/or PA stimulation are indicated by numbers. **B** The relative intensities of p-p70 S6K (T389), Lck (Y394) and STAT2 (Y689) sites. **C** Immunoblot of p-p70 S6K (Thr389) and its upstream molecular mTOR after insulin and/or PA treatment. **D** Expression level of mTOR and GRP78 under insulin and/or PA treatment with mTOR inhibition. Non-specific siRNA or mTOR siRNA was transfected into Ishikawa cells for 48 h first. Insulin and/or PA was then added into the treated cells. **E** Immunoblot of GRP78 and CHOP under insulin and PA combined treatment with or without pre-treatment of ATF6α inhibitor Ceapin-A7 (6 μM), PERK inhibitor ISRIB (0.2 μM) and IRE1 inhibitor 4μ8C (50 μM). **F** Schematic model of insulin enhancing PA-induced ER stress in EC cells

We further detected the changes in signalling pathways caused by insulin and/or saturated fatty acids via a phospho-kinase array. Ishikawa cells were treated with 100 nM insulin and/or 0.5 mM PA for 3 h, and among all the phosphorylation sites, the threonine residues (Thr389) of p70S6K were found to be upregulated most significantly. The results showed that insulin and PA alone increased the phosphorylation of p70S6K (Thr389), and PA combined with insulin upregulated p-p70S6K (Thr389) more significantly (Fig. 5A and B). As p70S6K (Thr389) could be directly phosphorylated by mTOR, we further confirmed the changes in mTOR/p70S6K (Thr389) by western blotting. The results showed that, after treatment with 100 nM insulin or 0.5 mM PA for 3 h or 6 h, Ishikawa cells expressed more mTOR protein and had higher p-p70S6K (Thr389) levels than vehicle-treated cells. Moreover, the increases in mTOR and p-p70S6K (Thr389) were more obvious with insulin combined with PA treatment than with insulin or PA treatment alone (Fig. 5C). We further used two specific siRNA to target mTOR to confirm the role of mTOR/p70S6K in insulin and PA-induced ER stress. In response to mTOR siRNA treatment in Ishikawa cells, GRP78 was obviously downregulated (Fig. 5D). We also found the expression level of insulin receptor (IR)-β was downregulated and p-AKT(Ser 473) increased under treatment (Additional file 1: Fig. S4C). However, glucose uptake had no change upon insulin and/or PA treatment (Additional file 1: Fig. S4D). These results indicated that saturated fatty acids synergised with insulin to activate the mTOR/p70S6K (Thr389) pathway and that mTOR/p70S6K mediate ER stress induced by saturated fatty acids combined with insulin in EC cells.

Metabolic-disorder-induced ER stress may activate UPR activation and thus be involved in metastasis and angiogenesis of EC. To dissect the role of three branches of UPR IRE1, PERK and ATF6 branches in EC cells, we detected the expression level of GRP78, under insulin and PA combined treatment with or without pre-treatment of ATF6α inhibitor Ceapin-A7 (6 μM), PERK inhibitor ISRIB (0.2 μM) and IRE1 inhibitor 4μ8C (50 μM). On the basis of the data, pre-treatment of ISRIB abolished GRP78 upregulation induced by insulin combined with PA. Also, the increased expression level of CHOP induced by insulin combined with PA decreased upon ISRIB pre-treatment. These results showed that insulin combined PA treatment induces ER stress and thus activates PERK-CHOP arm of the UPR in Ishikawa cells, which may result in EC progression (Fig. 5E).

In summary, the mTOR/p70S6K pathway plays an important role in metabolic-disorder-induced ER stress and, in turn, promotes UPR activation.

## Discussion

In this study, we demonstrated that patients with EC complicated with glucose metabolism disorders and dyslipidaemia were inclined to have prognostic high-risk factors for EC, such as lymphovascular space invasion, lymph node metastasis and greater tumour size, which may be attributed to hyperinsulinaemia and saturated fatty acid-induced UPR activation, Ca^2+^ homoeostasis imbalance and apoptosis in EC cells via mTOR/p70S6K-mediated ER stress (Fig. 5F).

Excessive insulin was not severe enough to induce apoptosis; however, it indeed enhanced the pro-apoptotic effect of saturated fatty acids on EC cells through ER stress and excessive mitochondrial Ca^2+^ uptake. ER stress plays a vital role in EC tumorigenesis and progression. Strong GRP78 predicted occult EC in complex atypical hyperplasia [28]. Moreover, GRP78 might be a prognostic indicator for EC, and high levels of GRP78 in visceral adipocytes indicated advanced-stage disease, deep myometrial invasion and poorer disease-free survival in patients with EC [29]. Our results, for the first time, demonstrated that ER stress was involved in metabolic abnormalities related to EC and mediated apoptosis in EC cells induced by insulin combined with saturated fatty acids.

We also found excessive Ca^2+^ entry into mitochondria upon ER stress induced by insulin combined with saturated fatty acids in EC cells. Mitochondrial Ca^2+^ overload activates enzymes such as α-ketoglutarate dehydrogenase, resulting in mitochondrial reactive oxygen species (ROS) overproduction [30]. Mitochondrial Ca^2+^ overload also induced opening of the mitochondrial permeability transition pore, mitochondrial membrane potential collapse and mitochondrial swelling, resulting in mitochondrial release of cytochrome C and cell apoptosis [30, 31]. We previously found that the cell membrane Ca^2+^ channel subunits Cav1.3 and TRPV4 and cellular Ca^2+^ influx promoted the proliferation and migration of EC cells [32, 33], and in the current study, we reported that excessive transfer of Ca^2+^ from the ER to mitochondria induced apoptosis of EC cells, demonstrating that homoeostasis of intracellular Ca^2+^ is important for EC progression.

Increased TG levels were related to impaired glucose metabolism in EC, consistent with increased APOB transcription in diabetic subjects with EC, revealing that glucose metabolism disorders accompany dyslipidaemia in patients with EC. Interestingly, obese patients with EC complicated with diabetes mellitus are prone to lymphovascular space invasion, lymph node metastasis and larger tumour sizes. Plasma apoB levels were positively correlated with postprandial TGs, and apoB led to dysfunction of white adipose tissue, resulting in decreased clearance of chylomicrons and insulin resistance [34]. Increased apoB was associated with an increased risk of type 2 diabetes [35, 36]. We found that APOB was the most significantly upregulated gene in diabetic patients with EC at the transcriptome level, confirming that higher apoB is related to insulin resistance in females. We propose that hypertriglyceridaemia and insulin resistance are likely to coexist in patients with EC with metabolic abnormalities.

Our results did not reveal the proliferative effect of insulin on Ishikawa, RL-952 and HEC-1B EC cells, even after excluding interference from FBS, and neither low- nor high-glucose conditions affected the results. However, we found that excessive insulin increased the ratio of Bax/Bcl-2 and induced DNA damage in EC cells. Insulin was reported to induce DNA damage in colon cancer cell lines, primary colon cells, peripheral lymphocytes and primary kidney cells in vitro and in rat kidneys in vivo through the overproduction of ROS [37, 38], and insulin-induced DNA damage in the endometrium has not yet been reported. Active p53 following DNA damage transcriptionally upregulates pro-apoptotic BH3-only subfamily members, which in turn neutralise the pro-survival proteins of the BCL-2 subfamily, and neutralisation of pro-survival BCL-2 family members is required for efficient apoptosis [25]. We thought that insulin induced the upregulation of pro-apoptotic proteins and the downregulation of anti-apoptotic proteins following DNA damage; however, the stress was not severe enough to induce cell apoptosis, and the cells survived and DNA damage accumulated to induce genome instability. We used EC cell lines as models of endometrial cells, and it would be more persuasive if the DNA damage induced by insulin was found in primary endometrial cells.

Our data showed that p70 S6K was activated by insulin combined with saturated fatty acids. mTOR was shown to directly phosphorylate S6K1 (Thr389), and S6K1 binds to and phosphorylates murine double minute 2 (Mdm2), preventing its translocation to the nucleus and inhibiting the ubiquitination of p53, resulting in an increase in p53-mediated DNA damage and DNA damage-induced cell apoptosis [39, 40]. PA has been reported to induce apoptosis in podocytes [41], cardiomyocytes [42] and hepatocytes [43]. For the first time, we proposed that saturated fatty acids induced apoptosis in the endometrium and that the pro-apoptotic effect was enhanced by insulin. Usually, apoptosis is considered to be tumour suppressive; however, it can potentiate tumour development as well. In dying cells, active caspase regulates downstream prostaglandin E2 (PGE2) release, which can potently stimulate the growth of neighbouring cells, promoting wound healing [44] and tumour repopulation in radiotherapy [45] or chemotherapy [46]. Apoptotic tumour cells not only promote coordinated tumour growth and angiogenesis but also induce tumour-promoting remodelling of macrophages in aggressive B-cell lymphomas [47]. Cell death triggers efferocytosis-induced wound-healing cytokines, including IL-4, IL-10, IL-13 and TGF-β, and promotes metastatic tumour progression in breast cancer [48]. Whether the apoptosis of EC cells induced by FFAs synergises with insulin to promote EC growth through PGE2 release or microenvironment remodelling remains to be further investigated. We demonstrated for the first time that excessive insulin and a high flux of saturated fatty acids, which are likely to co-exist in individuals with EC with metabolic abnormalities, synergistically induced UPR activation, Ca^2+^ homoeostasis imbalance and apoptosis in EC cells through ER stress.

## Conclusions

In summary, our data shed new light on the mechanism of metabolic disorders promoting EC. Our data demonstrated that excessive insulin boosted saturated fatty acid-induced UPR activation, Ca^2+^ homoeostasis imbalance and apoptosis through ER stress. This finding provides a rationale for targeting excessive insulin or ER stress to slow down metabolic disorder-mediated EC progression.

## Supplementary Information

**Additional file 1: Table S1.** Demographic characteristics of EC Cases. **Fig S1.** Serum lipids in Endometrial cancer patient with diabetes mellitus or higher BMI. A-B. The levels of LDL (*P* = 0.193) and CHO (*P* = 0.262) in diabetes and non-diabetes EC patients. C-F. The levels of TG (*P* = 0.064), HDL (*P* = 0.076), LDL (*P* = 0.459) and CHO (*P* = 0.507) in BMI ≥ 30 kg/m^2^ and BMI < 30 kg/m^2^ EC patients. G-H. The correlation of BMI and serum lipids. TG and HDL were positively correlated to BMI. The correlation coefficient r and P-value are shown. **Fig S2.** Differentially expressed genes (DEGs) analysis in Endometrial cancer patient with or without diabetes mellitus. A. Gene Ontology (GO) analysis of DEGs in EC with and without diabetes. The brighter the colour, the more significantly enriched GO term displayed. B-C. Expression levels of APOA2 and APOC3 in EC patients with or without diabetes. In the violin plot, the red dot represents the median, the red line represents the 95% confidence interval. D. mRNA expression level in tissues of EC patients with or without diabetes. *P < 0.05. **Fig S3.** Cell proliferation after insulin treatment. A. Proliferation of Ishikawa, HEC-1B and RL952 cells after treating with different doses (0, 1, 10, 100 nM) of Insulin for 0-72 h. B. Proliferation of Ishikawa cells after different concentrations of FBS (0%, 1%, 2.5%, 5% and 10%) treatment with or without Insulin. **Fig S4.** Apoptotic effect of insulin on EC cells. A. Apoptosis of Ishikawa and HEC-1B cells detecting by Annexin V-FITC/PI labeling flow cytometry after Insulin treatment. B. Immunoblot of Bax, Bcl-2, and β-actin after insulin treatment. Ishikawa cells are treated with 0.5 mM PA or 0.5 mM SA alone, and PA or SA plus 100 mM Insulin for 12 h. Curve shows Bax/Bcl-2 intensity relative to vehicle. C. Immunoblot of IR-β and p-AKT (S473) after insulin or/and PA treatment. D. Glucose uptake, indicating by fluorescence signals of 2-NBDG staining, after insulin or/and PA treatment.

## Abbreviations

EC: Endometrial cancer
ER: Endoplasmic reticulum
4-PBA: 4-Phenylbutyric acid
GO: Gene Ontology
FIGO: International Federation of Gynecology and Obstetrics
UPR: Unfolded protein response
