# Supplementary material for: Metabolic disorders sensitise endometrial carcinoma through endoplasmic reticulum stress
Source: Cell Mol Biol Lett. 2022 Dec 16;27:110. doi: 10.1186/s11658-022-00412-x (PMC9756454; doi:10.1186/s11658-022-00412-x)
Supplement: Supplementary file 1 — Additional file 1: Table S1. Demographic characteristics of EC Cases. Fig S1. Serum lipids in Endometrial cancer patient with diabetes mellitus or higher BMI. A-B. The levels of LDL (P = 0.193) and CHO (P = 0.262) in diabetes and non-diabetes EC patients. C-F. The levels of TG (P = 0.064), HDL (P = 0.076), LDL (P = 0.459) and CHO (P = 0.507) in BMI ≥ 30 kg/m2 and BMI < 30 kg/m2 EC patients. G-H. The correlation of BMI and serum lipids. TG and HDL were positively correlated to BMI. The correlation coefficient r and P-value are shown. Fig S2. Differentially expressed genes (DEGs) analysis in Endometrial cancer patient with or without diabetes mellitus. A. Gene Ontology (GO) analysis of DEGs in EC with and without diabetes. The brighter the colour, the more significantly enriched GO term displayed. B-C. Expression levels of APOA2 and APOC3 in EC patients with or without diabetes. In the violin plot, the red dot represents the median, the red line represents the 95% confidence interval. D. mRNA expression level in tissues of EC patients with or without diabetes. *P < 0.05. Fig S3. Cell proliferation after insulin treatment. A. Proliferation of Ishikawa, HEC-1B and RL952 cells after treating with different doses (0, 1, 10, 100 nM) of Insulin for 0-72 h. B. Proliferation of Ishikawa cells after different concentrations of FBS (0%, 1%, 2.5%, 5% and 10%) treatment with or without Insulin. Fig S4. Apoptotic effect of insulin on EC cells. A. Apoptosis of Ishikawa and HEC-1B cells detecting by Annexin V-FITC/PI labeling flow cytometry after Insulin treatment. B. Immunoblot of Bax, Bcl-2, and β-actin after insulin treatment. Ishikawa cells are treated with 0.5 mM PA or 0.5 mM SA alone, and PA or SA plus 100 mM Insulin for 12 h. Curve shows Bax/Bcl-2 intensity relative to vehicle. C. Immunoblot of IR-β and p-AKT (S473) after insulin or/and PA treatment. D. Glucose uptake, indicating by fluorescence signals of 2-NBDG staining, after insulin or/and PA treatment. [file 11658_2022_412_MOESM1_ESM.docx]

**Additional file 1: Table S1. Demographic characteristics of EC Cases**

| Variables(n=295) | Mean ± SD or n (%) |
| --- | --- |
| Age | 56.96±10.31 |
| BMI |  |
| Missing | 23(7.80) |
| ≥30kg/m2 | 42(14.24) |
| <30kg/m2 | 230(77.96) |
| Diabetes |  |
| YES | 64(21.69) |
| NO | 231(78.31) |
| Hypertension |  |
| YES | 114(38.64) |
| NO | 181(61.36) |
| Menopause |  |
| Missing | 1(0.34) |
| YES | 201(68.13) |
| NO | 93(31.53) |
| FIGO Stage |  |
| Missing | 26(8.81) |
| Early stage (FIGO I/II) | 222(75.26) |
| Advanced stage (FIGO III/IV) | 47(15.93) |
| Histologic subtype |  |
| EEC | 207(70.17) |
| NEEC | 88(29.83) |
| Fasting plasma glucose (mmol/L) | 6.07±1.65 |
| Triglyceride (mmol/L) | 1.43± 0.66 |
| Cholesterol (mmol/L) | 4.80±1.00 |
| Low-density-lipoprotein (mmol/L) | 2.99±0.81 |
| High-density-lipoprotein (mmol/L) | 1.23±0.30 |
| BMI, body mass index; FIGO, International Federation of Gynecology and Obstetrics ;EEC, endometrioid endometrial carcinoma;NEEC, non-endometrioid endometrial carcinoma; | |

**Additional file 1: Fig. S1. Serum lipids in Endometrial cancer patient with diabetes mellitus or higher BMI.** A-B. The levels of LDL (P=0.193) and CHO (P=0.262) in diabetes and non-diabetes EC patients. C-F. The levels of TG (P=0.064), HDL (P=0.076), LDL (P=0.459) and CHO (P=0.507) in BMI≥30kg/m^2^ and BMI<30kg/m^2^ EC patients. G-H. The correlation of BMI and serum lipids. TG and HDL were positively correlated to BMI. The correlation coefficient r and P-value are shown.

**Additional file 1: Fig. S2. Differentially expressed genes (DEGs) analysis in Endometrial cancer patient with or without diabetes mellitus.** A. Gene Ontology (GO) analysis of DEGs in EC with and without diabetes. The brighter the colour, the more significantly enriched GO term displayed. B-C. Expression levels of APOA2 and APOC3 in EC patients with or without diabetes. In the violin plot, the red dot represents the median, the red line represents the 95% confidence interval. D. mRNA expression level in tissues of EC patients with or without diabetes. *P<0.05

**Additional file 1: Fig. S3. Cell proliferation after insulin treatment.** A. Proliferation of Ishikawa, HEC-1B and RL952 cells after treating with different doses (0, 1, 10, 100nM) of Insulin for 0-72h. B. Proliferation of Ishikawa cells after different concentrations of FBS (0%, 1%, 2.5%, 5% and 10%) treatment with or without Insulin.

**Additional file 1: Fig. S4. Apoptotic effect of insulin on EC cells.** A. Apoptosis of Ishikawa and HEC-1B cells detecting by Annexin V-FITC/PI labeling flow cytometry after Insulin treatment. B. Immunoblot of Bax, Bcl-2, and β-actin after insulin treatment. Ishikawa cells are treated with 0.5mM PA or 0.5mM SA alone, and PA or SA plus 100mM Insulin for 12h. Curve shows Bax/Bcl-2 intensity relative to vehicle. C. Immunoblot of IR-β and p-AKT (S473) after insulin or/and PA treatment. D. Glucose uptake, indicating by fluorescence signals of 2-NBDG staining, after insulin or/and PA treatment.
